# Age-dependent increase of cytoskeletal components in sensory axons in human skin

**DOI:** 10.3389/fcell.2022.965382

**Published:** 2022-11-01

**Authors:** Klara Metzner, Omar Darawsha, Mengzhe Wang, Nayana Gaur, Yiming Cheng, Annekathrin Rödiger, Christiane Frahm, Otto W. Witte, Fabiana Perocchi, Hubertus Axer, Julian Grosskreutz, Monika S. Brill

**Affiliations:** ^1^ Department of Neurology, Jena University Hospital, Jena, Germany; ^2^ Institute of Neuronal Cell Biology, Technical University Munich, Munich, Germany; ^3^ Laboratory Animal Centre, Institute of Biomedicine and Translational Medicine, University of Tartu, Tartu, Estonia; ^4^ Helmholtz Diabetes Center (HDC), Helmholtz Center Munich, Institute for Diabetes and Obesity, Munich, Germany; ^5^ Munich Cluster of Systems Neurology (SyNergy), Munich, Germany; ^6^ Precision Neurology of the University of Lübeck, Lübeck, Germany; ^7^ PMI Cluster, University of Lübeck, Lübeck, Germany

**Keywords:** aging adults, human skin biopsy, sensory axon, actin, microtubule, neurofilament

## Abstract

Aging is a complex process characterized by several molecular and cellular imbalances. The composition and stability of the neuronal cytoskeleton is essential for the maintenance of homeostasis, especially in long neurites. Using human skin biopsies containing sensory axons from a cohort of healthy individuals, we investigate alterations in cytoskeletal content and sensory axon caliber during aging *via* quantitative immunostainings. Cytoskeletal components show an increase with aging in both sexes, while elevation in axon diameter is only evident in males. Transcriptomic data from aging males illustrate various patterns in gene expression during aging. Together, the data suggest gender-specific changes during aging in peripheral sensory axons, possibly influencing cytoskeletal functionality and axonal caliber. These changes may cumulatively increase susceptibility of aged individuals to neurodegenerative diseases.

## 1 Introduction

Aging is a complex process accompanied by disruptions in cellular homeostasis, the accrual of mutations, an increase of oxidative stress, and inflammatory processes (Mattson and Magnus, 2006; Doshi et al., 2010; Barth et al., 2019). Aging is further associated with a high degree of inter-individual heterogeneity in gene expression (Isildak et al., 2020). Both intrinsic and extrinsic factors, e.g., cellular atrophy and environmental influence, can drive the stochastic accumulation of genetic and epigenetic changes (Somel et al., 2006; Isildak et al., 2020; Puizina-Ivic, 2008; Zhang and Duan, 2018). While age-associated changes by themselves do not necessarily manifest in pathological phenotypic alterations or functional aberrations (Stahon et al., 2016), they may increase the risk for neurodegenerative disease (Hou et al., 2019; Wegmann et al., 2019). Moreover, there is an increasing piece of evidence that sex differences play an important role in physiological aging (Gur and Gur, 2002; Hagg and Jylhava, 2021). Sex difference is also manifested in the susceptibility ratios for neurodegenerative diseases. Men are more susceptible to neurodegenerative diseases: (McCombe and Henderson, 2010; Ullah et al., 2019) for instance, a male: female ratio of 2.5 and 1.4 in younger and aged individuals, respectively, has been reported for amyotrophic lateral sclerosis (ALS) (Manjaly et al., 2010).

The cytoskeleton is essential for the maintenance of cellular homeostasis and providing physical support, and particularly so in neurons, which feature extensive dendrites and specialized synapse-bearing axons for neurotransmission (Kapitein and Hoogenraad, 2015; Kevenaar and Hoogenraad, 2015; Konietzny et al., 2017; Kounakis and Tavernarakis, 2019; Munoz-Lasso et al., 2020). The cytoskeleton comprises neurofilaments, microtubules, and actin microfilaments. Neurofilament polypeptides are neuron-specific intermediate filaments, subdivided into three subunits based on chain length: light (NfL), medium (NfM), and heavy chain (NfH). Neurofilament fibers regulate axonal caliber, thereby indirectly affecting electrical conduction velocity (Sakaguchi et al., 1993; Kriz et al., 2000; Costa et al., 2018). Neurofilament assembly and function is primarily influenced by post-translational modifications including phosphorylation and/or glycosylation (Dong et al., 1993; Perrot et al., 2008; Yuan et al., 2012). Additionally, microtubules form a dynamic network that is constantly assembled and disassembled from α- and β-tubulin dimers on the distally growing plus-tipped end of microtubules (Gardner et al., 2014; Brouhard and Rice, 2018). The tubulin family contains several isotypes with type-III β-tubulin (TUBB3) being exclusively expressed in neurons (Katsetos et al., 2003a; Katsetos et al., 2003b). The microtubule network and associated motor proteins provide the basis for organelle and vesicle transport (Kapitein and Hoogenraad, 2015; Kevenaar and Hoogenraad, 2015). Microtubules can be ‘coded’ and assigned to different cellular functions *via* the differential expression of tubulin genes, post-translational modifications, and the differential recruitment of microtubule-associated proteins (e.g., tau) (Bulinski, 2019; Janke and Magiera, 2020). Finally, actin microfilaments are composed of globular (G-) actin monomers which polymerize within a dynamic system to filamentous (F-) actin (Korobova and Svitkina, 2010). Actin network assembly is involved in axon growth, shape-maintenance and synaptic signaling (Spence and Soderling, 2015; Kounakis and Tavernarakis, 2019; Lai and Wong, 2020). Further, the actin cytoskeleton contributes to cell motility and division, and intracellular transport (Svitkina, 2018; Kounakis and Tavernarakis, 2019). In the axon shaft, actin, together with spectrin, forms periodic ring-like structures to provide an additional stabilizing effect (Xu et al., 2013). Further complexity within the neuronal cytoskeleton results from the interplay of its components. Microtubules interact with the actin cytoskeleton (Dugina et al., 2016) to promote cellular adhesion processes (Rodriguez et al., 2003; Small and Kaverina, 2003). Moreover, microtubule dynamics may be affected by neurofilament binding (Bocquet et al., 2009). Indeed, increased neurofilament levels were shown to destabilize axonal microtubules in mice (Yadav et al., 2016). This interaction might be particularly relevant in neurodegenerative diseases, where elevated neurofilament levels have been observed in extra-neuronal liquids, including serum and cerebrospinal fluid, possibly owing to their resistance to proteolytic degradation (Zucchi et al., 2020; Yuan and Nixon, 2021).

Alterations of the neuronal cytoskeleton during aging are not well understood. While the deterioration of axonal transport with aging points to cytoskeletal dysregulation, the molecular mechanisms underlying this phenomenon remain to be fully elucidated. (Milde et al., 2015; Mattedi and Vagnoni, 2019). Dismantled microtubule structures have been previously reported in the human cortex, wherein soluble tubulin and microtubular density decreased in an age-dependent manner (Yan et al., 1985; Cash et al., 2003). The cytoskeletal aging phenotype in neurons might also differ between the central and peripheral nervous systems, owing to differences in the number and density of microtubules and neurofilament fibers (Saitua and Alvarez, 1988; Caselli et al., 1999).

Here, we aimed to investigate and distinguish between physiological and pathological cytoskeletal alterations that occur in neurons during aging. Our experimental paradigm focused on peripheral sensory nerve endings in human skin, as these fibers extend long processes that originate from somata located in the dorsal root ganglion next to the spinal cord (Rice and Albrecht, 2008). Proximal (thigh) and distal (ankle) punch biopsies are easily accessible and afford the opportunity to study sensory axon endings with different distances to the soma. By combining quantitative immunofluorescence stainings for cytoskeletal mass, measurements for axon caliber, and fiber density counts with transcriptomic data from skin tissue, we provide a comprehensive profiling of aging-associated changes in the expression of cytoskeleton-related genes.

## 2 Materials and methods

### 2.1 Participants and skin biopsy collection

All participants included in this study were recruited at the Jena University Hospital, Germany, between 2012 and 2014. They provided informed written consent, and the project was approved by the local ethics committee (no. 3369-02/12). Exclusion criteria were as follows: presence of: i) diabetes mellitus or other metabolic conditions, ii) neuromuscular diseases, iii) acute infections with fever, iv) alcohol abuse, v) tumors, and vi) coagulation disorders, smoking of more than five cigarettes daily, intake of oral coagulants (e.g., phenprocoumon), and allergy to local anesthetics. In total, 84 healthy study volunteers ranging from 23 to 79 years were included.

Skin biopsies were obtained by 3 mm punches (Stiefel Laboratories, STIEF-BP3, 22651) at Jena University Hospital. Three biopsies were performed under local anesthesia (mibe GmbH Arzneimittel, Xylocitin^®^-loc, lidocaine hydrochloride, 10 mg per punch site) at the proximal (thigh) and distal (ankle) leg, around 10 cm–15 cm above knee and ankle, respectively. Proximal skin biopsies used for histology were fixed in Zamboni’s solution [3.4% (v/v) paraformaldehyde, 15% (v/v) picric acid, in phosphate-buffered saline (PBS)] at 4°C for 48 h, then incubated in 10% (w/v) and 30% (w/v) sucrose for 24 h each. After freezing, biopsies were sectioned at 50 µm thickness using a cryostat (Leica CM3050S) and stored in anti-freeze storage solution (30% (v/v) ethylene glycol, 50% (v/v) Na_2_HPO_4_/NaH_2_PO_4_ phosphate buffer pH 7.4, 0.02% (w/v) sodium azide, 15% (w/v) glucose) at −20°C.

Cohorts were split based on sex (51 males and 21 females) and individuals were age-matched. These were further binned into 4-age-group charts for visualization and analyses: 20s (24–29 years old, 9 male, 6 female individuals), 40s (45–50 years old, 13 male, 5 female), 60s (60–64 years old, 14 male, 5 female), and 70s (74–79 years old, 15 male, 5 female).

### 2.2 Assessment of fiber density values

Skin sections were washed in Tris-buffered saline (TBS) and incubated in 1.5% (v/v) hydrogen peroxide (H_2_O_2_) in TBS/0.1% (v/v) Triton X-100 for 30 min. After two washing steps in TBS and blocking in blocking solution [TBS, 0.1% (v/v) Triton X-100, 3% (v/v) donkey serum, 2% (w/v) bovine serum albumin (BSA), 2% (w/v) milk powder] for 30 min, skin sections were incubated in anti-Protein Gene Product (PGP) 9.5 antibody (Abcam, ab72911, 1:1,000) diluted in blocking solution at 4°C overnight. Sections were washed again in TBS and incubated in a biotinylated donkey anti-mouse secondary antibody (Dianova, 715-065-151, 1:500) for 2 h at room temperature. Following washing with TBS, skin sections were incubated with avidin-biotin reagent [TBS/0.1% (v/v) Triton X-100; Vector Laboratories, VECTASTAIN^®^ Elite ABC-HRP Kit, Peroxidase (Standard), PK-6100] for 1 h at room temperature, and washed again. Staining was performed with 3,3′-Diaminobenzidine solution [TBS/0.1% (v/v) Triton X-100; Sigma-Aldrich, D4293] and H_2_O_2_ [TBS/0.1% (v/v) Triton X-100; Sigma-Aldrich, U8879] for 12 min. After washing, sections were mounted on glass slides using a 0.5% (w/v) gelatin solution.

Slides were washed with distilled water and incubated with hematoxylin solution (Sigma-Aldrich/Fluka, 51260) for 1 min. After another washing step in warm water, sections were incubated in eosin solution (Carl Roth, 7,089.1) for 1 min. Slides were washed with water, isopropanol solutions [80% (v/v) once, 100% (v/v) twice], and Neo-Clear^™^ (Sigma-Aldrich, 1.09843). Mounting of slides was performed using Neo-Mount^™^ mounting medium (Sigma-Aldrich, 1.09016) and High Precision cover glasses (170 μm ± 5 μm, No. 1.5H, 24 × 50 mm; Marienfeld, 0107222).

Brightfield microscopy for routine diagnostic purposes was used to count intraepidermal nerve fiber density (IENFD) defined by the number of stained nerve fibers crossing the basal lamina of the skin divided by the length of the epidermal surface. ImageJ V1.47 (National Institutes of Health, United States) was used for image processing (Schneider et al., 2012).

### 2.3 Immunostaining and analysis

Skin sections were washed in PBS and incubated at 4°C overnight in the following primary antibodies: anti-Neurofilament H, Nonphosphorylated Antibody (BioLegend, 801701, 1:1,000), Alexa Fluor^®^ 488 or 594 anti-Tubulin Beta 3 (BioLegend, 657404/657408, 1:400), anti-PGP9.5 Polyclonal Antibody (Invitrogen^™^, PA1-10011, 1:200), anti-Gelsolin antibody (Abcam, ab11081, 1:200), diluted in blocking solution [10% (v/v) normal goat serum, 1% (w/v) BSA, 0.2% (v/v) Triton X-100, in PBS]. Following washing in PBS for 20 min, sections were incubated in secondary antibody (goat anti-mouse IgG1 Alexa Fluor^®^ 594 or 488, Invitrogen™, A-21125/A-21121, 1:1,000), Alexa Fluor^®^ 647 Phalloidin (Invitrogen^™^, A-22287, 1:400), and Hoechst 33342 (Invitrogen™, H1399, 1:1,000) in blocking solution for 1 h at room temperature. After a final washing step in PBS for 1 h, sections were mounted on glass slides using Fluoroshield^™^ (Sigma-Aldrich, F6182) and High Precision cover glasses. Staining fidelity was confirmed using negative controls omitting the primary antibody (Supplementary Figure S1A–F).

Three image stacks from different skin biopsy sections were acquired using a confocal microscope (Olympus FV1000, 20X/0.8 N.A. air-, 60X/1.42 N.A. oil-immersion objective). In total, we analyzed 30–60 single axons for the Mean Gray Value (representing the average gray value within the image selection, defined as the sum of gray values of all selected pixels divided by the total number of pixels). The Mean Gray Values were corrected for background signal by subtracting the mean intensity of 3 regions outside the skin tissue. Axon diameter was analyzed in the composite image (merge of single images of the three channels acquired from the triple staining for NfH, TUBB3, and phalloidin) by either 1) approximating a vertical line in the axon, or 2) by measuring the area and the length of this axonal region and dividing one by the other. This was performed with the open-source software Fiji by ImageJ V1.53 (National Institutes of Health, United States) (Schindelin et al., 2012). Images presented on figures are maximum intensity projections of 10–15 images each. All samples were processed and analyzed with the experimentalist blinded to provenance.

### 2.4 Statistical analysis

Statistical analyses and graphical representation were performed using GraphPad Prism (V9.3.1 for Windows, GraphPad Software). Between-group differences in fiber density, axon diameter, and cytoskeletal protein content were evaluated using a Kruskal–Wallis test, with Dunn’s correction for multiple comparisons. A two-way analysis of variance (ANOVA), with Tukey’s correction for multiple comparisons, was performed to assess group differences within the diameter distribution analysis. Points on all graphs represent one individual. Statistical significance was set as follows: *p < 0.05, **p < 0.01, ***p < 0.001, ****p < 0.0001.

### 2.5 Analysis of differentially expressed genes from RNA-sequencing data of skin tissue

The raw RNASeq fastq data was first published by (Aramillo Irizar et al., 2018) and retrieved from NCBI’s Gene Expression Omnibus (GSE75337, GSE103232). Transcriptomic data was only available from the skin tissue of the male individuals. The reads were aligned to the human reference genome (Ensembl genome version 101) using STAR 2.7.6a (Dobin et al., 2013). The quality was checked with FastQC (http://www.bioinformatics.babraham.ac.uk/projects/fastqc) and Qualimap 2 (Okonechnikov et al., 2016). The low-quality base pairs and adapters were trimmed with Trimmomatic (Bolger et al., 2014). The transcripts per million (TPM) values were quantified with RSEM (Li and Dewey, 2011). Genes were retained when the maximum of the mean TPM values across different age groups was greater than 5 and the maximum of the fold change across any two groups was greater than 1.5. Cytoskeletal genes were obtained based on the UniProt subcellular annotation. Gene Ontology (GO) analysis was performed using DAVID Bioinformatics Resources (Huang et al., 2008; Huang et al., 2009) after dividing the cytoskeletal genes into two groups with increasing and decreasing expression with age.

## 3 Results

### 3.1 Fiber density in skin to exclude neuropathies

**FIGURE 1:**
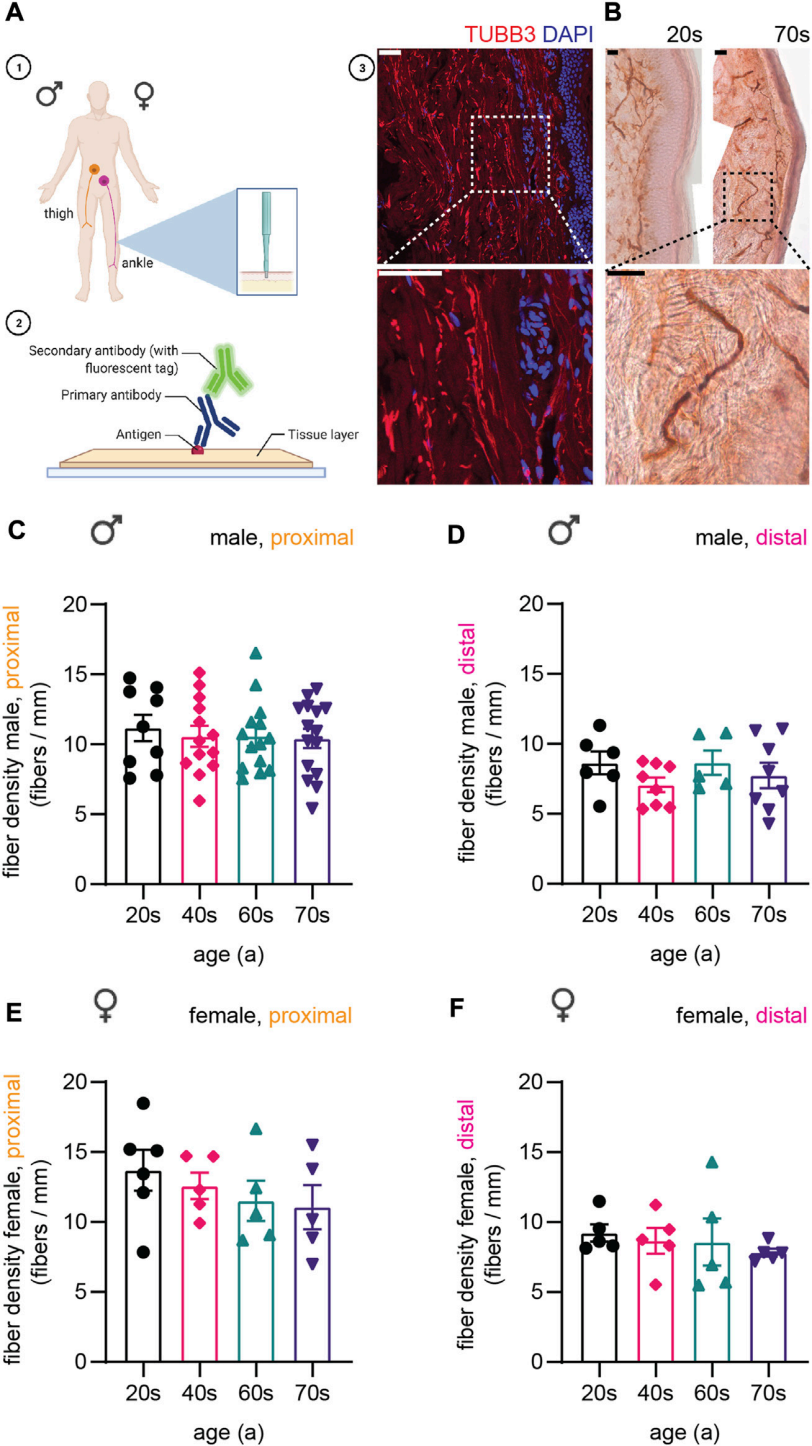
Display of experimental procedure and quantification of sensory nerve fiber density. **(A-1)** Schematics of skin biopsy location: proximal (thigh) and distal (ankle) region of the leg. **(A-2)** Skin sections immunostained for cytoskeletal proteins. Created with BioRender.com. **(A-3)** Confocal image of a skin biopsy section stained for type III β-tubulin (TUBB3, red) and DAPI (nuclear stain, blue). **(B)** Images for immunohistochemical staining for PGP9.5 of two individuals of the youngest (20s, left) and the oldest (70s, right) age group. Higher magnification of boxed area shows a single fiber. **(C–F)** Quantification of IENFD for **(C)** male proximal, **(D)** male distal, **(E)** female proximal, and **(F)** female distal sample sets. One data point in **(C–F)** represents the IENFD value of one individual. Data represent mean ± SEM. Scale bars in **(A-3)** and **(B)** are 50 µm.

**FIGURE 2:**
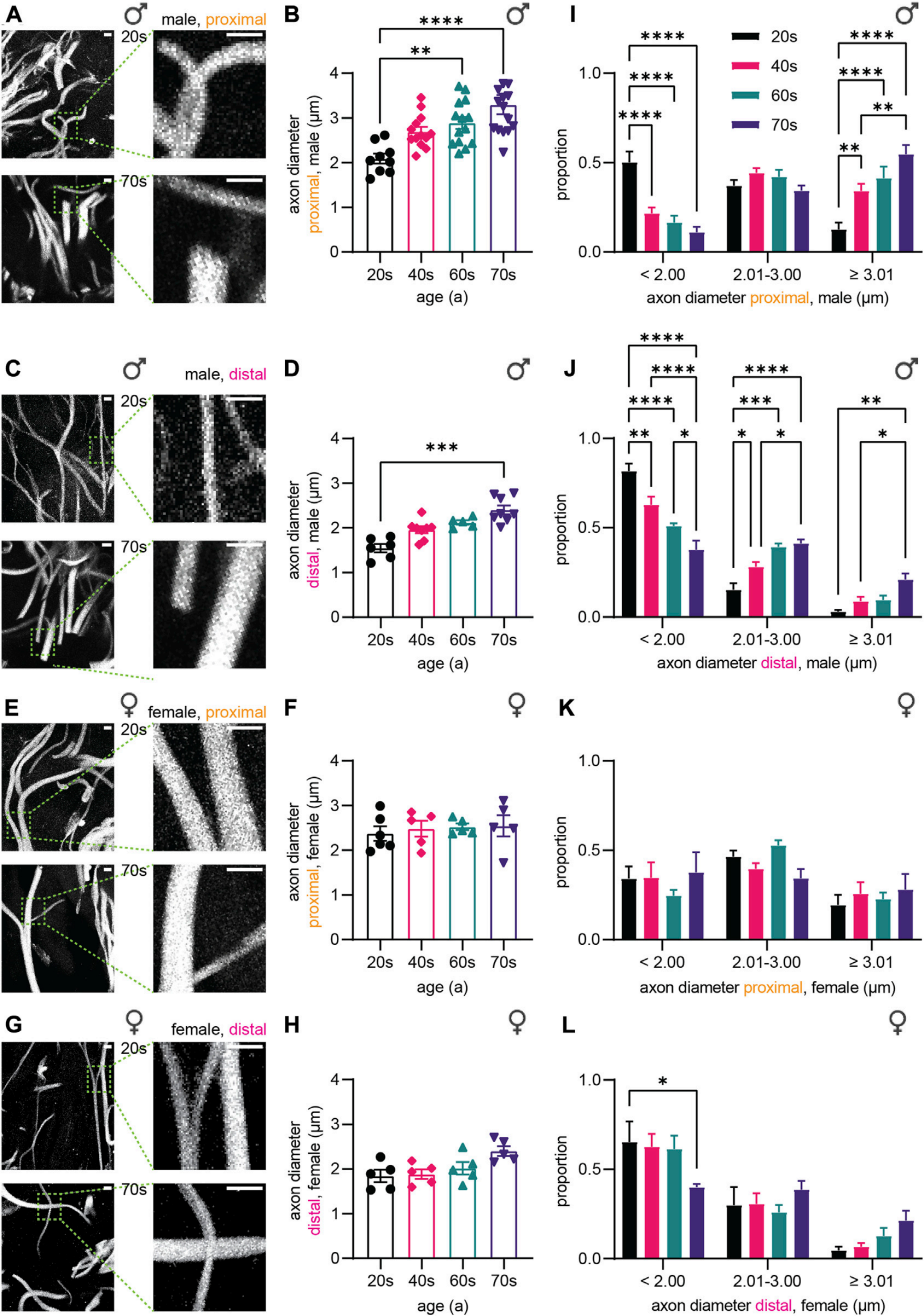
Quantitative analysis of the axonal diameter.**(A,C,E,G)** Confocal images of TUBB3 immunostaining (white) of skin biopsies from **(A)** male proximal, **(C)** male distal, **(E)** female proximal, and **(G)** female distal leg. **(B,D,F,H)** Graphs depict axon diameter (µm) in **(B)** male proximal, **(D)** male distal, **(F)** female proximal, and **(H)** female distal skin biopsies (3 images per individual, ≤ 20 axons per image). **(I–L)** Axon diameter distribution for the **(I)** male proximal, **(J)** male distal, **(K)** female proximal, and **(L)** female distal sample sets. Diameter spans are defined as follows: < 2.00 µm, 2.01 ≤ … < 3.00 µm, ≥ 3.01 µm. Scale bars in **(A,C,E,G)** are 5 µm. Points in graphs **(B,D,F,H)** symbolize one individual. Data represent mean ± SEM. **(B,D,F,H)** Kruskal–Wallis test with Dunn’s multiple comparisons test. **(I-L)** Two-way ANOVA with Tukey’s multiple comparisons test. *p < 0.05, **p < 0.01, ***p < 0.001, ****p < 0.0001.

**FIGURE 3:**
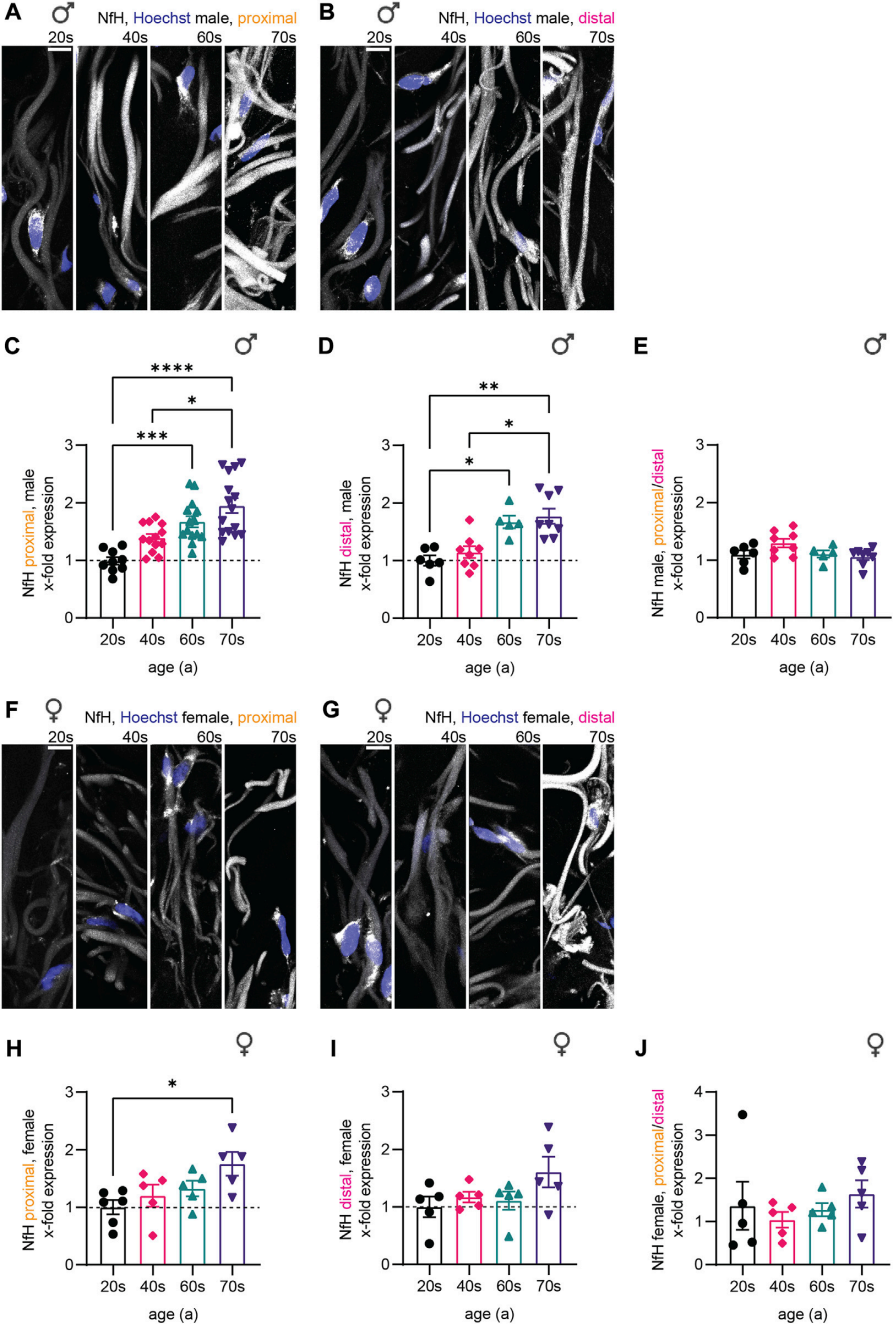
Quantitative immunostaining of human skin sections for NfH. **(A,B,F,G)** Confocal images of immunostaining for NfH (white) with nuclear stain (Hoechst, blue) for one individual of each age group (from left to right: 20s, 40s, 60s, and 70s) are shown for the **(A)** proximal male, the **(B)** distal male, the **(F)** proximal female, and the **(G)** distal female sample set. **(C,D,H,I)** Quantification of NfH intensity in sensory axons (x-fold normalized to average of 20s group) (3 images per individual, ≤ 20 axons per image, **(C)** *n* = 51 individuals in proximal male, **(D)** *n* = 27 in distal male, **(H)** *n* = 21 in proximal female, **(I)** *n* = 20 in distal female data). **(E,J)** Differences between the male and female proximal and distal samples were determined by calculating the NfH expression ratio for each individual. Scale bars in **(A,B,F,G)** are 10 µm. Points in graphs **(C–E,H–J)** represent on individuals. Data represent mean ± SEM. Kruskal–Wallis test with Dunn’s multiple comparisons test. *p < 0.05, **p < 0.01, ***p < 0.001, ****p < 0.0001.

In order to analyze changes in the composition of neuronal cytoskeletal components during physiological aging, we sampled skin biopsies from the proximal (thigh) and distal (ankle) region of the leg from healthy individuals aged 23–79 years (Figure 1A-1, criteria to be included into the study see *Materials and Methods*). Fixed skin biopsies were sectioned, immunostained (Figure 1A-2), and image stacks quantitatively analyzed for fiber density, axon diameter, or intensity (Figure 1A-3). Skin biopsies from healthy males and females were split into four groups: 20s (24–29 years old), 40s (45–50 years old), 60s (60–64 years old), and 70s (74–79 years old).

In a first step, we quantified intraepidermal nerve fiber density (IENFD) based on immunohistochemical staining for Protein Gene Product 9.5 (PGP9.5, Figure 1B), which is widely used as the gold standard in clinical practice to assess IENFD from skin sections (Van Acker et al., 2016). Proximal fiber counts (male proximal on average: 10.6 ± 2.6 fibers/mm, female proximal on average: 12.3 ± 3.1 fibers/mm) were overall higher than distal fiber counts (male distal on average: 7.9 ± 2.1 fibers/mm, female distal on average: 8.6 ± 2.1 fibers/mm) [Figure 1C–F, (McArthur et al., 1998)]. We observed a decrease in IENFD with age in the male proximal (on average: 20s: 11.2 ± 2.7 fibers/mm, 40s: 10.6 ± 2.6 fibers/mm, 60s: 10.6 ± 2.5 fibers/mm, 70s: 10.4 ± 2.6 fibers/mm), female proximal (on average: 20s: 13.7 ± 3.6 fibers/mm, 40s: 12.6 ± 2.1 fibers/mm, 60s: 11.5 ± 3.2 fibers/mm, 70s: 11.1 ± 3.5 fibers/mm), and female distal samples (on average: 20s: 9.2 ± 1.2 fibers/mm, 40s: 8.7 ± 1.9 fibers/mm, 60s: 8.6 ± 3.4 fibers/mm, 70s: 7.8 ± 0.6 fibers/mm) as described previously (McArthur et al., 1998; Lauria et al., 2010), but not for the male distal sample set (Figure 1C–F). The data confirmed the absence of neuropathic conditions affecting the structure and number of sensory nerve fibers in skin and served as basis for the following immunostaining experiments.

### 3.2 Sensory axon caliber increases during aging are only evident in males

Next, we measured axon diameters and their distribution in our cohort of skin biopsies (Figure 2), according to age, gender, and location. We found a drastic age-dependent increase in caliber of sensory nerve endings in male proximal and distal skin biopsies (Figure 2A–D). Average axon diameter increased 1.6-fold from 2.1 µm ± 0.3 µm (proximal) and 1.5 µm ± 0.2 µm (distal) for individuals in their 20s up to 3.3 µm ± 0.8 µm (proximal) and 2.4 µm ± 0.3 µm (distal) in their 70s (Figure 2B,D). Significance was reached in sensory axons at around age 60 in proximal samples compared to the 20s group (20s: 2.1 µm ± 0.3 µm, 60s: 2.9 µm ± 0.5 µm, *p* = 0.0059, Figure 2B), while in distal samples axon thickness was significantly altered at around 70s (20s: 1.5 µm ± 0.2 µm, 70s: 2.4 µm ± 0.3 µm, *p* = 0.0002, Figure 2D). Together, neurites with longer distance from the soma are overall thinner, but diameter increased in a similar manner with age in male samples. Strikingly, in proximal and distal female skin biopsies (Figure 2E–H), the diameter of sensory axons remained similar in all four age groups assessed (proximal: 20s: 2.4 µm ± 0.4 µm, 40s: 2.5 µm ± 0.4 µm, 60s: 2.5 µm ± 0.2 µm, 70s: 2.5 µm ± 0.5 µm, Figure 2F; distal: 20s: 1.8 µm ± 0.3 µm, 40s: 1.9 µm ± 0.2 µm, 60s: 2.0 µm ± 0.3 µm, 70s: 2.4 µm ± 0.2 µm, Figure 2H), pointing to sex-specific aging effects in sensory axons.

In order to determine if the increase in axon diameter is a general phenomenon or restricted to a few axons, we analyzed the distribution of axon thickness. According to the overall average of 2.5 µm (all groups), we binned small axons < 2 μm, 2 µm–3 µm thick axons, and large axons > 3 µm. The number of ‘average’-sized axons (2 µm–3 µm thickness) remained similar in all four age groups of proximal male biopsies. In contrast, large axons (> 3 µm) were rare amongst the 20s samples, but increased during aging in proximal (Figure 2I) and distal male skin biopsies (Figure 2J) from 13% (proximal) and 3% (distal) to 55% (proximal) and 21% (distal), respectively. Concomitantly, the proportion of thin axons (< 2 µm) decreased in male skin biopsies [20s: 50% (proximal), 82% (distal), 70s: 11% (proximal), 38% (distal)]. In female biopsies, the distribution of axon diameter remained similar during aging, in agreement with the similar average axon thickness (Figure 2K,L). Hence, the proportion of large (> 3 µm) and small (< 2 µm) sensory axons in all groups did not vary significantly for the proximal samples from the female individuals [20s: 19% (large), 34% (small), 70s: 28% (large), 38% (small), Figure 2K]. The distal female biopsies showed a significant decrease in small (<2 µm) sensory axons from the 20s to the 70s (20s: 65%, 70s: 40%, *p* = 0.0334), which seems to be compensated by slight increases in the proportion of thicker axons in the 70s age group (Figure 2L). Taken together, our data demonstrate that the majority of axons in males undergo caliber increase during aging, and the change of sensory axon caliber strongly depends on sex.

### 3.3 Age-dependent increase in cytoskeletal components in sensory nerve endings

In the following experiments, we focused on cytoskeletal protein levels in sensory axons during aging. Following immunostainings of skin biopsy sections for a subunit of neurofilaments (NfH), neuron-specific tubulin (TUBB3), and F-actin (phalloidin), we recorded confocal image stacks, analyzed the immunostaining intensities within axonal regions, and compared the relative amount of protein levels. Graphs represent normalized intensity on the average of the youngest age group (20s, x-fold, see *Materials and methods* for details).

NfH immunostaining intensity increased strongly during aging as shown by representative confocal images of the dermis (Figures 3A,B,F,G). In male proximal biopsies, the quantitative analysis revealed a 1.7-fold increase from the 20s until the 60s age group (Figure 3C) with similar effects in distal skin biopsies (Figure 3D). Here, significance was reached in the 60s age group (proximal: *p* = 0.0006, distal: *p* = 0.0419). NfH protein levels steadily increased until 1.8-fold and 1.4-fold in the oldest age group for the female proximal and distal samples, respectively. Notably, the only statistical significance in the female proximal samples was reached between the 20s and 70s age group (*p* = 0.0465) while the increase in the female distal samples was not statistically significant (20s–70s: *p* = 0.6141), which could either indicate a smaller or delayed increase of NfH levels in women (Figure 3H, I). To determine whether axon length affects NfH levels increase during aging, we calculated the ratio of proximal to distal intensities for the respective individuals (Figure 3E, J). Indeed, male and female individuals revealed no significant length-dependent change in NfH levels. In order to corroborate specificity of NfH immunostaining and to exclude autofluorescence artifacts, we performed control stainings by omitting primary antibodies (Supplementary Figure S1A–F) and assessed PGP9.5 immunostaining intensity on a sub-cohort of 21 male skin biopsies (Supplementary Figure S1G). While we found a significant increase from the 20s to the 40s age group (*p* = 0.0110), PGP9.5 intensity was not changed during aging in the 40s, 60s and 70s (Supplementary Figure S1H), confirming specific increase in NfH content during physiological aging independent of sex and location.

**FIGURE 4:**
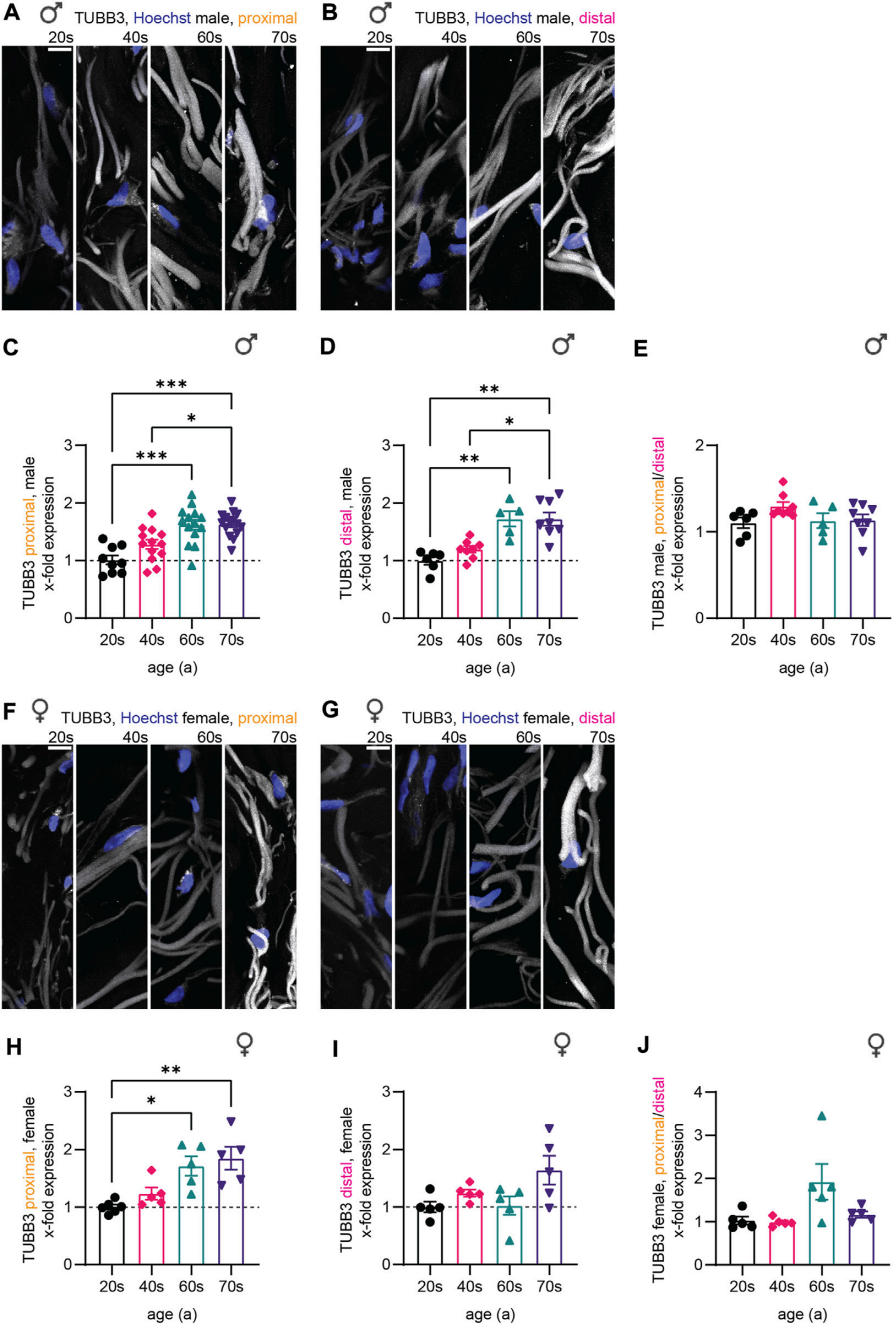
Quantitative immunostaining of human skin sections for TUBB3. **(A,B,F,G)** Confocal images of human skin biopsies immunostained for TUBB3 (white) with nuclear stain (Hoechst, blue) taken from individuals of the four age groups (20s, 40s, 60s, and 70s) from **(A)** male proximal, **(B)** male distal, **(F)** female proximal, and **(G)** female distal samples. **(C,D,H,I)** Quantification of TUBB3 gray values in sensory axons (x-fold normalized to average of 20s group) (3 images per individual, ≤ 20 axons per image, **(C)** *n* = 51 individuals in proximal male, **(D)** *n* = 27 in distal male, **(H)** *n* = 21 in proximal female, **(I)** *n* = 20 in distal female data). **(E,J)** Male and female proximal and distal sample sets were compared *via* the proximal/distal TUBB3 expression ratio. Scale bars in **(A,B,F,G)** are 10 µm. Points in graphs **(C,D,H,I)** represent on individuals. Data represent mean ± SEM. Kruskal–Wallis test with Dunn’s multiple comparisons test. *p < 0.05, **p < 0.01, ***p < 0.001.

Similar to NfH, microtubular mass (measured with TUBB3 immunostaining intensity) in sensory nerve endings increased during aging, again independent of sex and biopsy location (Figures 4A,B,F,G). Quantitative analysis for the male sub-cohort revealed a significant increase in TUBB3 levels from the 20s until the 60s age group (1.7-fold for both proximal and distal), which stagnated in the oldest individuals (70s, Figure 4C,D). The results were similar for the female individuals (proximal: 20s–70s: 1.8-fold increase; distal: 20s–70s: 1.5-fold increase) (Figure 4H,I). As the proximal/distal intensity ratio was not significantly different between the four age groups, we conclude that age-related physiological changes occur independent of axon length (Figure 4E,J), pointing to a general increase in protein of cytoskeletal compounds, rather than a single component changing.

**FIGURE 5:**
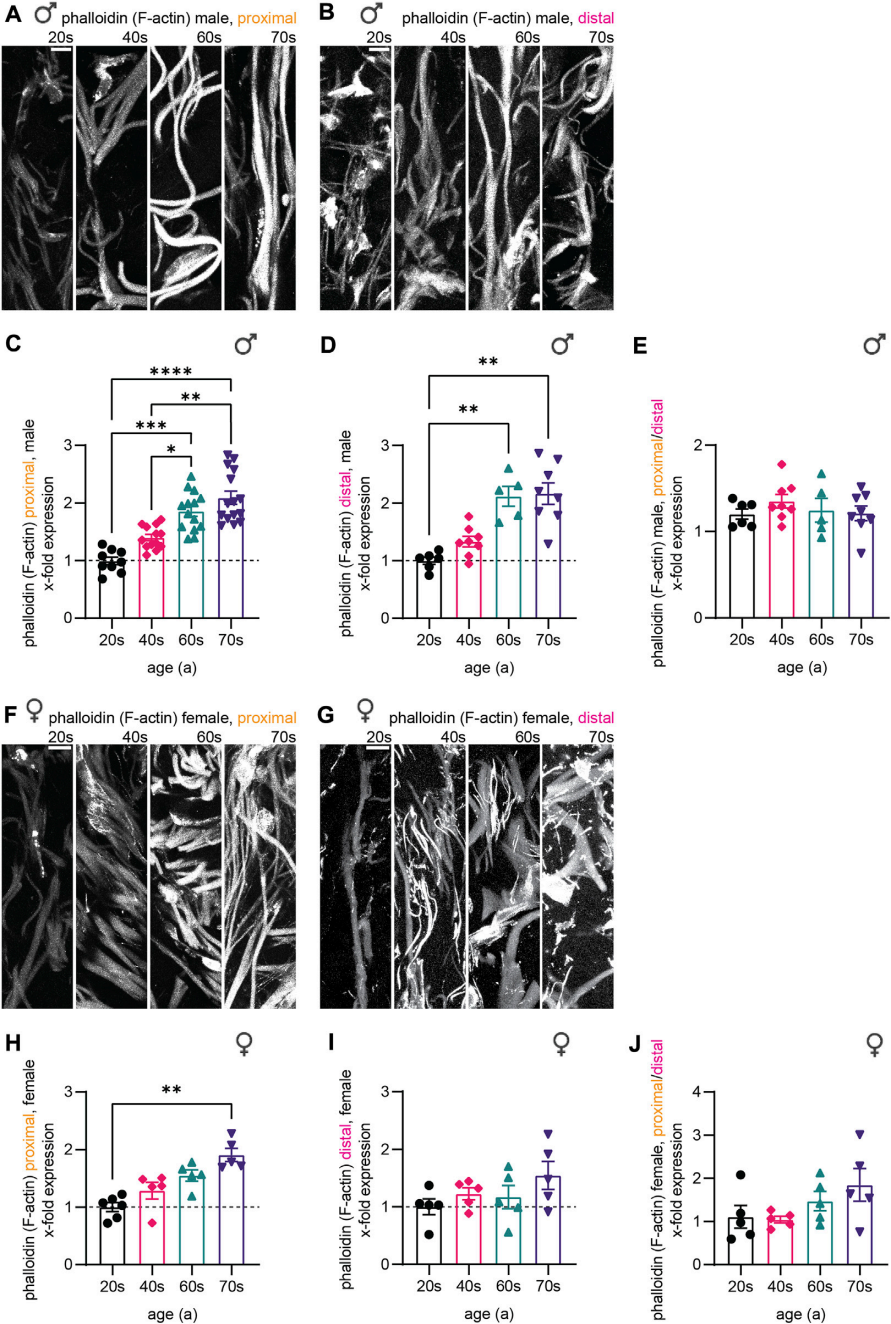
Quantitative immunostaining of human skin sections for phalloidin. **(A,B,F,G)** Phalloidin labeling (white) for the **(A)** male proximal, the **(B)** male distal, the **(F)** female proximal, and the **(G)** female distal samples of the four age groups (20s, 40s, 60s, and 70s). **(C,D,H,I)** Quantitative analysis confirmed these results for each respective data set (x-fold normalized to average of 20s group) (3 images per individual, ≤ 20 axons per image, **(C)** *n* = 51 individuals in proximal male, **(D)** *n* = 27 in distal male, **(H)** *n* = 21 in proximal female, **(I)** *n* = 20 in distal female data). **(E,J)** Phalloidin intensity ratio between the male and female proximal and distal samples allowed to compare both sample sets. Scale bars in **(A,B,F,G)** are 10 µm. Points in graphs **(C,D,H,I)** represent on individuals. Data represent mean ± SEM. Kruskal–Wallis test with Dunn’s multiple comparisons test. *p < 0.05, **p < 0.01, ***p < 0.001, ****p < 0.0001.

Finally, we assessed F-actin levels, which we visualized using a phalloidin staining (Figures 5A,B,F,G). Similar to microtubules and neurofilaments, gray values resembling F-actin increased with age in the male sample sets during aging (2.1-fold for proximal and distal) and plateaued from the individuals in their 60s towards those in their 70s (Figure 5C,D). In the female proximal samples, F-actin levels revealed a 1.9-fold increase without stagnation from the 60s to the 70s (Figure 5H). This increase was less profound in female distal biopsies (1.3-fold increase from 20s to 70s, Figure 5I). Also, F-actin levels were not significantly different corresponding to nerve fiber length when comparing the proximal and the distal samples (Figure 5E,J).

Together, all three markers we analyzed—neurofilaments, microtubules, and F-actin—revealed an increase in protein levels during aging for male and female individuals. While protein levels of the three components increased evenly for the female individuals (females proximal—NfH: 1.8-fold, TUBB3: 1.9-fold, F-actin: 1.9-fold), F-actin levels were disproportionately elevated in male skin biopsies implying an altered composition of the cytoskeleton in sensory nerve fibers in males with aging (males proximal—NfH: 1.7-fold, TUBB3: 1.7-fold, F-actin: 2.1-fold). Furthermore, in the oldest age group (74-79a), the levels for males plateaued compared to the 60s group, while female samples showed steady increase. In combination with caliber changes, cytoskeletal changes could lead to discrepancies specifically in the oldest age group in males, which could possibly result in a homeostatic misbalance within the axons.

### 3.4 Age-dependent alterations in cytoskeletal gene expression in human skin

To verify if the observed changes of the cytoskeletal protein composition are specific to sensory neurites or represent a general cellular phenomenon in human skin, we next analyzed previously performed RNA sequencing of the skin biopsies of male individuals of the underlying cohort (Aramillo Irizar et al., 2018), which our stained samples were a subset from. While this data set has been previously analyzed for the link of cancer and degenerative diseases (Aramillo Irizar et al., 2018), senescence and inflammation (Barth et al., 2019), and the circadian system (Barth et al., 2021) in regard to aging, we now focused on genes related to cytoskeletal components (see *Materials and Methods* for details).

**FIGURE 6:**
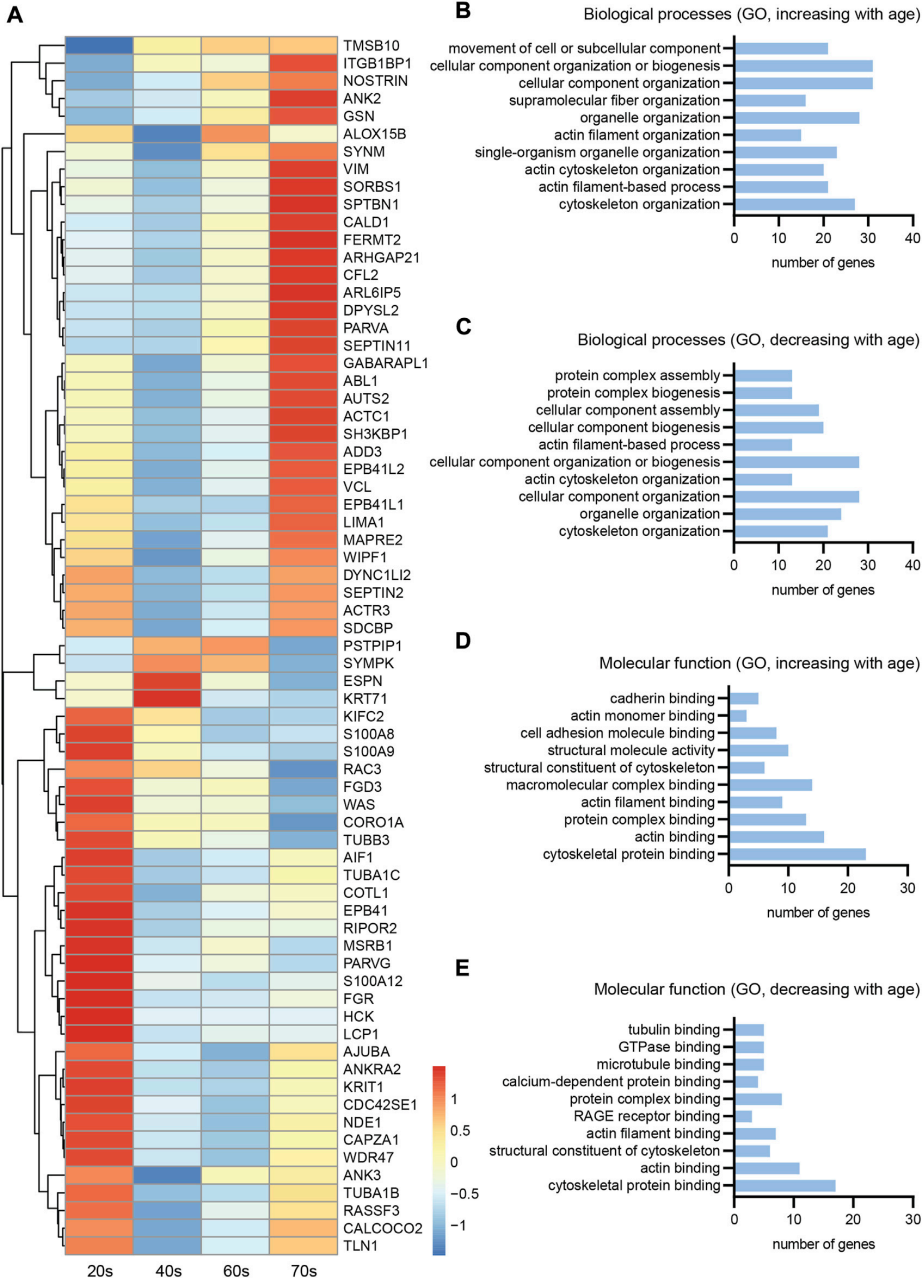
Transcriptomics of skin biopsies for cytoskeletal gene expression. **(A)** Normalized row-scaled expression of transcriptomic data filtered for differentially expressed cytoskeletal genes between the four age groups (20s, 40s, 60s, and 70s). Main expression patterns: an increase with age (**A**, top), an increase towards the 40s/60s with a following decrease (**A**, middle), and a decreasing expression with age (**A**, bottom). **(B–E)** Gene Ontology (GO) analysis for the cytoskeletal genes, divided into two groups with either increasing or decreasing expression with age, regarding both biological processes (**B**, increasing; **C**, decreasing) and molecular function (**D**, increasing; **E**, decreasing), revealing many of the differentially expressed cytoskeletal genes to be associated with the actin cytoskeleton.

Analysis of the transcriptomic data set revealed three patterns of differential expression of cytoskeletal genes in relation to aging (Figure 6A). Cytoskeletal gene expression steadily increased with aging (Figure 6A, top), whereas another pattern indicated a decrease in gene expression levels with age (Figure 6A, bottom). The third, less abundant pattern had increased gene expression levels until peaking in the middle age groups (40s/60s), then a steady decrease until the highest age group (Figure 6A, middle). Given the low number of genes belonging to the latter group, aberrant expression of cytoskeletal genes seems to either predominantly increase or decrease with age. The initial trend of an expression increase or decrease during young adulthood appears to manifest with increasing age. The low transcript counts of neuron-specific microtubule- [average transcripts per million (TPM) value of all age groups for TUBB3: 5.4] and intermediate filament (average TPM value of all age groups for NfH: 0.1)-associated genes, though not surprising, prevented detailed analysis of their expression levels during aging.

**FIGURE 7:**
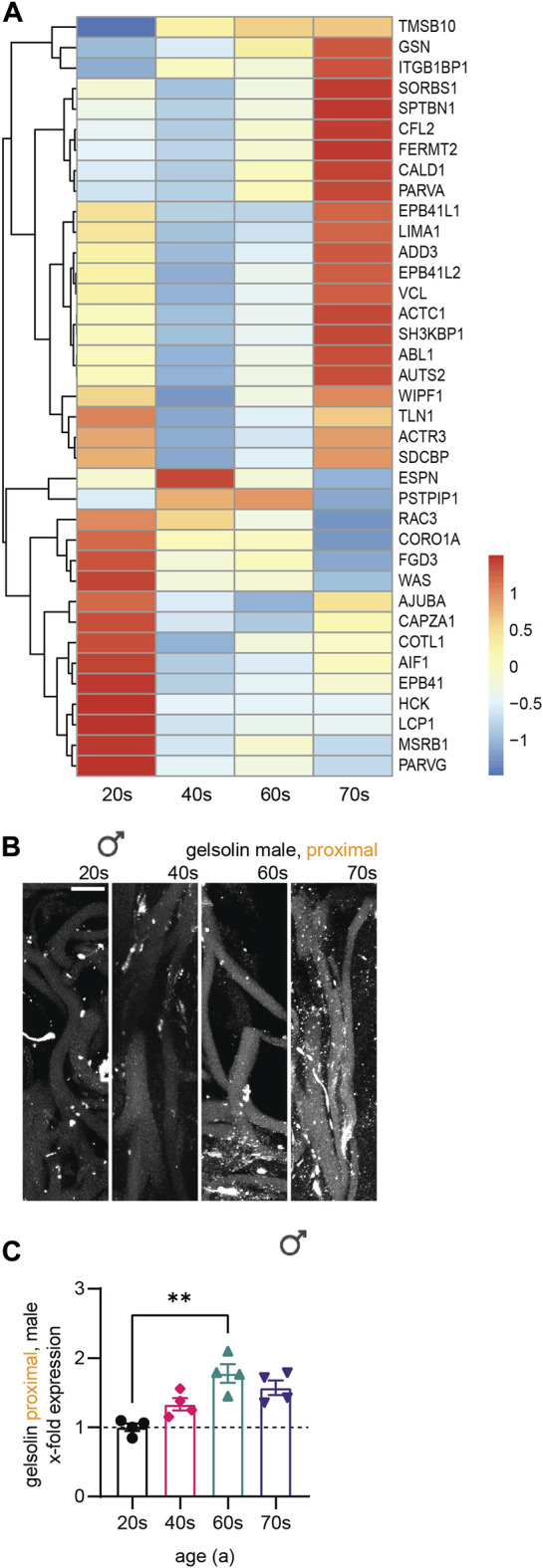
Association of differentially expressed cytoskeletal genes with the actin cytoskeleton. **(A)** Normalized row-scaled expression of transcriptomic data filtered for differentially expressed cytoskeletal genes associated with the actin cytoskeleton as identified by GO analysis (Figures 6B–C) between the four age groups (20s, 40s, 60s, and 70s). **(B)** Gelsolin immunostaining (white) for a subset of male proximal skin sections (*n* = 11). **(C)** Quantification of gelsolin gray values in sensory axons (x-fold normalized to average of 20s group) (3 images per individual, 10 axons per image). Scale bar in **(B)** is 10 µm. Points in graph **(C)** represent on individuals. Data represent mean ± SEM. Kruskal–Wallis test with Dunn’s multiple comparisons test.

To further classify the differentially expressed cytoskeletal genes within skin tissue (Figure 6A), we divided them into two groups showing either increasing or decreasing expression with age and conducted Gene Ontology (GO) analysis (Figure 6A)regarding associated biological processes (increasing, Figure 6B; decreasing, Figure 6C) and molecular functions (increasing, Figure 6D; decreasing, Figure 6E). This analysis revealed a high proportion of the proteins encoded by the cytoskeletal genes to be associated with the actin cytoskeleton, independent of whether gene expression increased or decreased with age. Actin-related genes were highly expressed, revealing a tendency of the respective genes to either increase or decrease with age (Figure 7A). However, RNA extraction from a skin biopsy punch is not axon-specific, but also included the surrounding skin tissue. To verify whether these differential expression results of whole skin stand on a single axonal level, we performed immunostainings for gelsolin on skin biopsy sections from a subset of eleven male proximal samples (Figure 7B). Gelsolin is an actin-binding protein, which regulates dynamics of actin filament assembly in sensory neurites as well as in surrounding cells (Heidings et al., 2020; Lee and Kang, 2020). Gelsolin protein levels significantly increased with age from the 20s to the 60s age group (1.8-fold increase, *p* = 0.0065, Figure 7C). Combined with previous immunofluorescence staining results of F-actin, we confirmed the increased expression with age that was observed by analyzing the transcriptomic data of skin tissue (Figure 6A). However, gelsolin was not only expressed in sensory neurites, but also in surrounding cells, which supports our approach of immunofluorescence stainings to visualize age-dependent changes in protein content.

## 4 Discussion

The present study analyzed cytoskeletal components in sensory axons in skin biopsies from healthy human donors in relation to age, sex, and neurite length. Our data indicates a general increase in the neurofilament, microtubule, and F-actin protein content during aging, with this trend appearing to be independent of sensory neurite length. Axonal caliber changes drastically in a sex-specific manner, as increases in diameter were only noted in male samples.

### 4.1 Skin biopsy as a versatile model for the study of biological aging

Cytoskeletal composition and organization is crucial for axonal structure and integrity (Kevenaar and Hoogenraad, 2015). Skin, as the most voluminous organ of the body and directly exposed to the outer environment, has naturally been the focus of many aging studies (Puizina-Ivic, 2008). Studying skin may also offer novel insights into the pathophysiology of complex multi-systemic neurodegenerative conditions with extra-motor features (Isaacs et al., 2007). Furthermore, skin biopsies are easily accessible *via* non-invasive procedures (Nischal et al., 2008). Nerve fiber densities of our participants were in the range of normal values reported by a global normative reference study (Figure 1) (McArthur et al., 1998; Lauria et al., 2010), indicating that skin biopsies were performed in accordance with best practices and yielded reliable results.

### 4.2 Neuronal cytoskeleton and aging

Our data suggest a general increase in cytoskeletal components, with the effect being more pronounced for F-actin relative to microtubules and neurofilament polypeptides (Figure 3–5). This increase may be indicative of an overall ‘ossification’ of axons with reduced flexibility. Ultrastructural analysis of the sciatic nerve in aging rats showed a similar increase in microtubule and neurofilament density (Saitua and Alvarez, 1988). Indeed, microtubule flow and fluctuation were recently found to be crucial for driving neuronal polarization during development (Schelski and Bradke, 2021). It may be possible that a similarly decreased retrograde flux during aging could lead to an increase in tubulin, as well as other cytoskeletal components, especially at the axon tips. Nevertheless, an increased microtubular mass does not directly relate to improved axonal transport. On the contrary, an abundance of cytoskeletal proteins may impair the axonal transport machinery (Côté et al., 1993). This may be due to the presence of dysfunctional subunits and fiber fragments as a result of impaired polymerization of microtubules and actin filaments. However, a dysfunctionality of the cytoskeleton regarding its dynamism and the polymerization of its subunits could not be assessed by our study.

Our data is in line with other studies on cytoskeletal changes in the context of aging. TUBB3 has been proposed as a biomarker for aging, as its levels were shown to increase in human keratinocytes (Lehmann et al., 2017). Whether this increase in cytoskeletal components is cell type-specific, and the mechanisms that drive this differential expression during aging remain to be clarified. Aging is broadly associated with increased heterogeneity of gene expression in the human brain (Isildak et al., 2020), possibly due to substantial epigenetic variations (Cheung et al., 2018). Our transcriptomic analysis (Figure 6) also confirms altered gene expression with age. Whether cytoskeletal protein upregulation is the result of increased gene expression or an impairment of degradation pathways, as a result of enhanced stability due to post-translational modifications for instance, remains to be elucidated (Didonna and Opal, 2019).

### 4.3 Sex-dependency of the neuronal cytoskeleton

Interestingly, our results showed a sex-specific effect; no age-associated nerve fiber caliber increase was observed in females (Figure 2). In contrast to the relative decrease in protein content (cytoskeletal protein levels stagnated while axon size increased) observed in elderly men, women in the same age group displayed an increase in relative cytoskeletal mass (cytoskeletal protein levels rose with stagnating axon diameter). These differences suggest that biological sex, in addition to age, may contribute to alterations in cytoskeletal protein levels. Indeed, sex hormones can influence the development of the human brain both with respect to the formation of synaptic structures and the neuronal cytoskeleton (Hansberg-Pastor et al., 2015). Sex hormone levels were also shown to affect the F-actin content of alveolar macrophages (Tsotakos et al., 2016). These sex-associated differences in cytoskeletal content changes may potentially contribute to the differential vulnerability to neurodegenerative disease in aging males and females (McCombe and Henderson, 2010; Ullah et al., 2019).

### 4.4 Actin cytoskeleton and aging

Here, we assessed F-actin levels by phalloidin staining, as this is a proxy for total actin levels. However, age-driven changes in F-actin content—as observed here—may also result from an altered F-actin/G-actin ratio rather than a change in total actin content. The increase and subsequent arrest of F-actin could have a positive impact on axon stability as well as our GO enrichment analysis (Figures 6B–E, Figure 7A) revealed that a significant proportion of the differentially expressed proteins were associated with the actin cytoskeleton. Transcriptomic analysis of skin showed that the actin-binding protein gelsolin was upregulated with increasing age. This was confirmed at the protein level *via* immunostaining of peripheral nerve fibers from skin sections (Figures 7B,C), thereby further validating our experimental paradigm. Whether β-actin expression increases or decreases during aging remains inconclusive, with some studies reporting increased F-actin content (Garcia and Miller, 2011) and conversely, decreased β-actin expression during aging in non-neuronal cells (Moshier et al., 1993; Li et al., 2017). Actin dynamics are also highly dependent on actin-binding proteins (Gourlay and Ayscough, 2005; Li et al., 2017). Additional experiments evaluating cytoskeletal component subtypes, the post-translational modification landscape, and associated proteins, may help shed light on age-associated alterations.

### 4.5 Link to age-related phenomena and pathologies

There is an increasing evidence that sensory perception is affected during aging. For instance, cutaneous sensitivity has been reported to decline with age (Bell-Krotoski et al., 1993), with perceptual thresholds also differing between sexes (Bowden and McNulty, 2013). Moreover, sensitivity towards low-intensity pain, particularly heat pain, deteriorates with age, (Lautenbacher et al., 2017). Although this age-associated decline in sensory perception may be linked to the altered cytoskeletal composition inside sensory fibers, fully exploring this is beyond the scope of this paper. Furthermore, pathological protein aggregation is a shared hallmark of several neurodegenerative diseases: for instance, β-amyloid peptides and tau in Alzheimer’s disease and transactive response DNA-binding protein 43 (TDP-43) in ALS (Phillip et al., 2015; Jo et al., 2020). Interestingly, actin-rich deposits like Hirano bodies have also been observed in ALS and Alzheimer’s disease (Rao and Cohen, 1990; Hirano, 1994; Gourlay and Ayscough, 2005). These aggregations may result from mRNA transport defects and a subsequently altered subcellular localization landscape and cellular stress (Canclini et al., 2020; Chin and Lecuyer, 2020; Markmiller et al., 2021). In addition, nucleocytoplasmic transport defects might affect this tight regulation of the exchange of proteins and RNAs between cytoplasm and nucleus (Giampetruzzi et al., 2019). Taken together, the observed age-related changes in the composition and structure of the neuronal cytoskeleton hold an important scientific value, as they may be related to the occurrence of neurodegenerative diseases.

### 4.6 Limitations

Here, we describe age-associated cytoskeletal alterations in peripheral sensory nerves. However, our results do not dissect the functionality or dynamism of the neuronal cytoskeleton during aging; further experiments to identify the mechanistic basis for the observed changes and how these may influence susceptibility to neurodegenerative diseases are therefore needed. Additionally, transcriptomic analysis was performed on whole skin tissue, which naturally includes several cell types in addition to sensory nerve fibers. Given that the cell bodies of these fibers are not located in the skin, our analysis may have only captured the RNA within the axon endings, which is relatively small to begin with and potentially further “diluted” by material from surrounding skin cells. As a result, the cytoskeletal alterations observed at the protein level cannot be directly ascribed to the gene expression changes noted in the transcriptomic data set. Rather, these may manifest as an epiphenomenon of the profound differential gene expression that occurs during aging. While single axon-sequencing may soon be feasible with the advent of novel technologies like axon sequencing, applicability is still restricted to *in vitro* models, thus precluding its integration within our experimental paradigm (Nijssen et al., 2018; Nijssen et al., 2019).

## Data Availability

The datasets presented in this study can be found in online repositories. The names of the repository and accession numbers can be found below: https://www.ncbi.nlm.nih.gov/, GSE75337 and GSE103232.

## Supplementary material

Supplementary Figure 1 Negative control staining and immunostaining for PGP9.5 and quantification. **(A–F)** Confocal images of the **(A–C)** sample staining (application of both primary and secondary antibody) and the **(D–F)** negative control (application of the secondary antibody with omission of the primary antibody). **(G,H)** Immunostaining for PGP9.5 (white) and quantification was performed on the proximal samples from 21 male individuals as an additional control. Graph represents mean values ± SEM. The scale bars represent 10 μm. **p* < 0.05.

## Abbreviations

ALS, amyotrophic lateral sclerosis; ANOVA, analysis of variance; BSA, bovine serum albumin; F-actin, filamentous actin; G-actin, globular actin; GO, Gene Ontology; H2O2, hydrogen peroxide; IENFD, intraepidermal nerve fiber density; NfH, neurofilament heavy chain; NfL, neurofilament light chain; NfM, neurofilament medium chain; PBS, phosphate-buffered saline; PGP9.5, Protein Gene Product 9.5; TBS, Tris-buffered saline; TDP-43, transactive response DNA-binding protein 43; TPM, transcripts per million; TUBB3, type-III β-tubulin.
