# Supplementary figures and images for: Age-dependent increase of cytoskeletal components in sensory axons in human skin
Source: Front Cell Dev Biol. 2022 Nov 1;10:965382. doi: 10.3389/fcell.2022.965382 (PMC9664158; doi:10.3389/fcell.2022.965382)

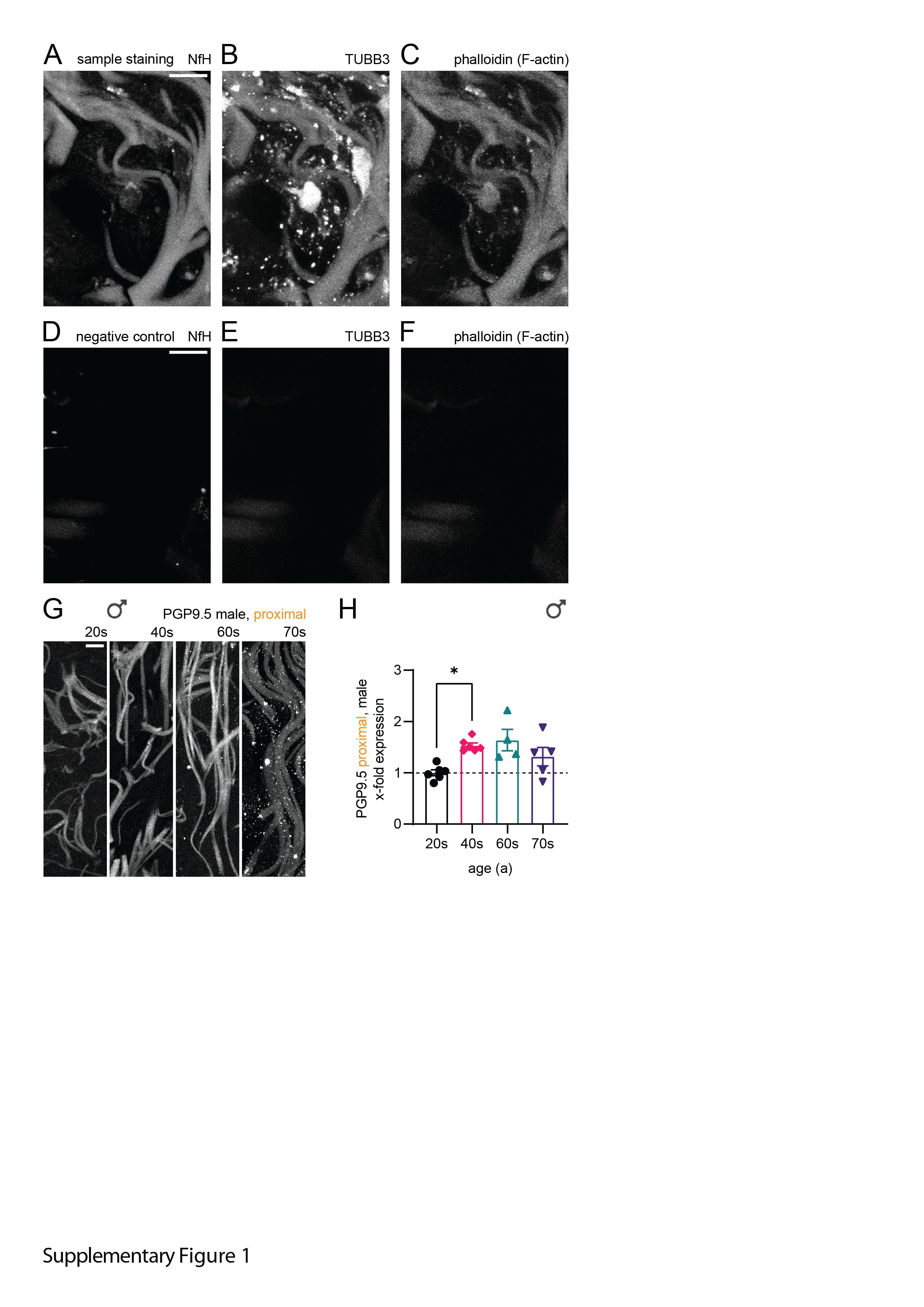

Supplement: Supplementary file 1 [file Image1.TIF]
